# A metamodel for heritage-based urban recovery

**DOI:** 10.1186/s43238-022-00068-8

**Published:** 2022-11-08

**Authors:** Christer Gustafsson, Matthias Ripp

**Affiliations:** 1 Department of Art History, Uppsala University (SE), Uppsala, Sweden; 2 Department of Archives & Preservation of Historical Monuments City of Regensburg (DE), Regensburg, Germany

**Keywords:** Urban heritage, Recovery, Resilience, Sustainable development, Metamodel, Conservation

## Abstract

**Purpose:**

The purpose of this paper is to discuss the potential transfer of a metamodel for heritage-based urban development (HBUD) in a postcrisis urban recovery scenario.

**Design/methodology/approach:**

After an introduction to the field of cultural heritage as a resource for urban development, the research question is elaborated, and the current understanding of urban heritage is explored. The use of the metamodel in a postcrisis urban recovery setting is described as a potential solution. The proposed metamodel is introduced along with the grounded theory and design research methodology through which it was developed. The specific qualities of metamodels and how they can contribute to the proposed use are highlighted. The scenario is then developed further, and specific ways in which the metamodel could contribute are elaborated. Finally, the metamodel is compared to other methods, such as the historic urban landscape (HUL) approach, and the limitations are discussed.

**Findings:**

The metamodel can potentially be used in a postcrisis urban recovery scenario. The metamodel cannot be used directly, owing to the nature of metamodels; however, it can be transferred to a specific context and help to structure successful heritage-based urban recovery (HBUR) processes.

**Practical limitations/implications:**

One limitation is that it can be difficult to understand the differences between models and metamodels. Only with a comprehensive understanding of the nature of metamodels can this metamodel be applied, for example, to select appropriate models for HBUR. The metamodel can help to ensure that all relevant ‘elements’ are part of the processes designed for HBUR and emphasise the need for thorough planning, or scoping, of such processes.

**Originality/value:**

Metamodelling has not previously been used for HBUD or HBUR.

## Introduction

During the current global crisis caused by the COVID-19 pandemic, several surveys have been conducted, and reports have determined what interventions and additional resources are needed for the recovery of global, national, regional, and urban economies (UNCTAD 2020; OECD 2021). Studies have also shown how the cultural heritage sector is affected by the crisis (Fears et. al. 2020; UNESCO 2020; European Commission 2020a, b, c; World Bank 2021). UNESCO published a series of reports on the impact of COVID-19 on the cultural sector (World Heritage, living heritage, cultural and creative industries, and museums) (UNESCO 2021a, 2021b, 2021c, 2021d) and, with the World Bank, a report on the impact on cities, culture and creativity (UNESCO and the World Bank 2021). ICOMOS conducted a survey on the impact of COVID-19 on heritage (tangible, intangible and natural), which resulted in insights and recommendations for building a more resilient heritage framework (ICOMOS 2020). In 2021, the European Commission published guidelines on the safe resumption of activities in the cultural and creative sectors.^1^ Europeana suggested that there is an opportunity for digital transformation and a basis for a common approach to capacity building for the recovery, resilience, and sustainability of the cultural heritage sector.^2^ In the UK, the Department for Digital, Culture, Media and Sport announced a rescue package to safeguard culture and heritage from the economic impact of COVID-19, and the National Lottery Heritage Fund in England is distributing governmental funding in partnership with Historic England.^3^ This initiative aims to ensure that heritage organisations can operate on a viable and sustainable basis.

However, the problem is that there is no general methodological framework for using cultural heritage for recovery after a crisis. This paper focuses on how cultural heritage can be *used as* a resource and a starting point for recovery. Cultural heritage should therefore be understood not only as something valuable that needs to be protected or restored but also as a resource that can be used for recovery and to improve the quality of life of local communities more generally.

In recent decades, cultural heritage has been discussed several times in relation to various crises, e.g., war, terrorism and conflicts, climate change, illegal trafficking, and unauthorised excavations and construction projects (World Bank 1998; Stanley-Price 2007; Barakat 2020). In parallel, there has been an increase in the literature and research on cultural heritage and resilience; however, this research has usually focused on risk assessment, risk preparedness, and generally how at-risk cultural heritage can be *protected from* crises and disasters (ICCROM and Canadian Conservation Institute 2016; O’Brien et al. 2015; Jigyasu 2016).

UNESCO’s Warsaw Recommendation on Recovery and Reconstruction of Cultural Heritage highlights the use of heritage in postconflict and postdisaster situations where the overall goal is the recovery of society (UNESCO 2018). A team of researchers from Italian and Spanish universities presented a model for cultural heritage life cycle management as an innovative methodology for the recovery and restoration of cultural heritage (Settembre Blundo et al. 2014). In this model, the environmental, economic, and social impacts of activities of recovery, restoration, and valorisation of cultural heritage were assessed and then applied to the case of recovery processes.

In the EU Interreg project ‘Culturecovery’, nine partners from six central European countries collected best practices of intangible cultural heritage recovery and preservation, including the possibility of transferring their methodologies to different circumstances, with the aim of providing guidelines for sustainable management. Focusing on ecomuseums, the project shared knowledge and experience from innovative management and financing for the preservation, recovery and sustainable and responsible use of intangible cultural heritage.

Daly and Rahmayati (2012) critically discussed the understanding of recovery as ‘building back better’, considering the relationship between change and recovery in postdisaster environments and the importance of cultural heritage within that relationship. Naomi Klein stressed the risk of government and corporate interests exploiting postconflict and postdisaster reconstruction processes for political or economic gain (Klein 2007).

Integrated conservation and cultural heritage already have a substantial and successful history of use as urban development resources and contributors to inclusive, sustainable, and innovation-driven development (Pereira Roders and van Oers 2011; Nocca 2017; UNESCO and the World Bank 2018). Consequently, after crises, heritage can be at the heart of urban economic, health, and social recovery strategies. Scientific publications on cultural heritage in relation to circular economies, creativity, innovation, smart cities, and resilience have begun to demonstrate an awareness of how these concepts can contribute to postcrisis recovery strategies.

## The starting point and problem: what tool can we use to activate heritage for urban recovery and stimulate resilience?

**Fig. 1:**
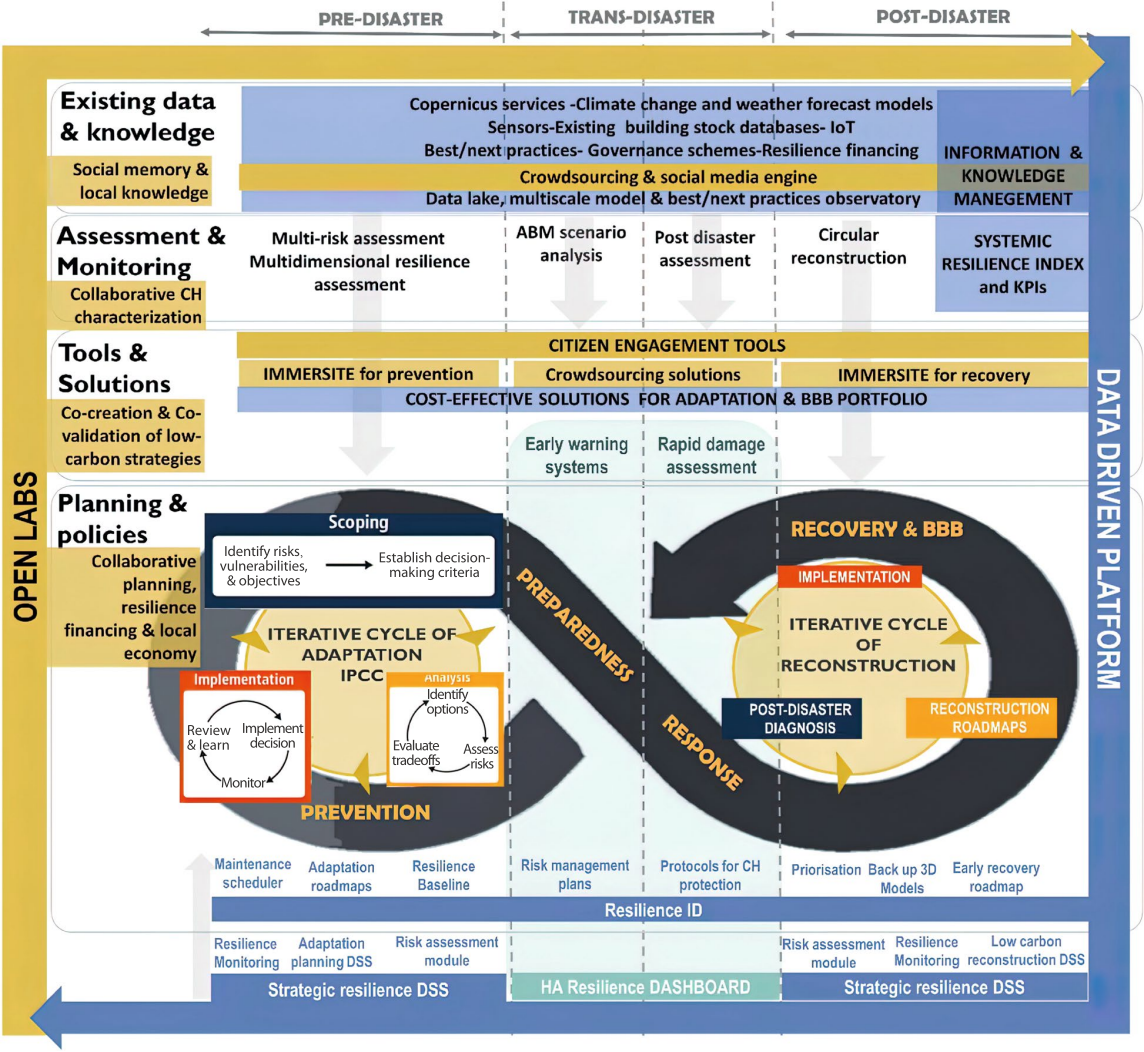
Resilience concept of the SHELTER project. (Source: SHELTER 2020B)

This paper proposes a universally applicable metamodel to design, evaluate, and improve heritage-based urban recovery (HBUR) processes. It is based on a compilation of documents, bibliographical research and desk study. Three successful models for cultural heritage as a driver of urban development are used as cases for this paper: the Heritage as Opportunity (HerO) project, the Community-Led Urban Strategies in Historic Towns (COMUS) project, and the Halland model project.^4^ These are analysed using grounded theory and design research methodology, and a metamodel for heritage-based urban development (HBUD) is designed. The paper elaborates on how this metamodel can be used in postcrisis urban recovery and development scenarios. The underlying concept of resilience is based on the Sustainable Historic Environments hoListic reconstruction through Technological Enhancement and community-based Resilience (SHELTER) project and its defined phases of response and recovery/build back better (see Fig. 1).

Our goal is to introduce metamodelling to the field of integrated conservation of cultural heritage and to develop a strategic understanding of its potential for HBUD and HBUR. The research question is as follows: how can such a metamodel contribute to enhancing the quality of life of local communities in postcrisis recovery scenarios? The concept of cultural heritage has been extensively discussed; for instance, the Council of Europe defined it ‘as a group of resources inherited from the past which people identify, independently of ownership, as a reflection and expression of their constantly evolving values, beliefs, knowledge and traditions. It includes all aspects of the environment resulting from the interaction between people and places through time’ (Council of Europe 2005, 2). Today, cultural heritage is recognised as a resource for sustainable development (United Nations 2015a, b) and an asset for regional development (European Commission 2014a; European Commission 2014a, 2018) as well as sustainable urban development (UNESCO 2013; United Nations 2016; European Commission 2017).

To help the reader understand this approach, the following subsections provide definitions of some terms that are used throughout the text. Among these definitions, it is particularly important to understand the differences between models and metamodels. They are fundamentally different concepts with different use scenarios, levels of abstraction, and limitations. We also want to be precise regarding where the introduced metamodel can be used, and for this purpose, it is important to be clear about the different phases of resilience to which we are referring. The differences and commonalities between urban development and recovery and regeneration, which are terms used in different contexts, are also clarified. This clarification is important for understanding the context and scenarios for which the metamodel is proposed. One limitation is connected to the inherent logic of metamodelling and is based on the selection of the source models from which the metamodel was developed. It must be acknowledged that a broader variety of models (in terms of geographical representation or different time frames) that were used as a basis could have led to different elements and thus a modified metamodel (Ripp 2021).

### Models

Models can accomplish two essentially diverse functions. A model can be an illustration of a selected part of the world, what Frigg and Hartmann call the ‘target system’ (Frigg and Hartmann 2018, 741), and it can stand for a theory since it interprets the laws and axioms of that theory. However, these two concepts are not, according to Frigg and Hartmann, simply understood from an either-or perspective, as scientific models can be representations in both senses at the same time. Several scientific models that represent a phenomenon can be understood as an umbrella term covering all relatively stable and general features associated with that phenomenon. The term ‘model’ is often used without communicating the method-inherent limitation of models to a specific context.

### Metamodels

The abstraction and logic levels in a metamodel are higher than those in a normal model, and the content of a metamodel can be developed from models (Van Gigch 1991, 256). For this purpose, the content of the models needs to be represented in more abstract ways. A metamodel cannot be directly used to find solutions for a specific problem or situation but can be used to select appropriate models and tools for specific situations or challenges. Metamodelling is often used in ecology, information science, and military decision making (Dobrovic 2001; Van Gigch 1991). Following the ideas of Van Gigch (1991), a metamodel is a model that is logically one level above a normal model. To describe this relation, Van Gigch also uses the term ‘inquiring systems’. According to his understanding, these are systems ‘devoted to the creation, acquisition, production and dissemination of knowledge’ (Van Gigch 2003, 3). The metamodel inquiry system is based on epistemology. A metamodel can also be used to assess and apply change, adaptation, and transformation and to restructure and enhance a system (Barile and Saviano 2015, 73). It is in the domain of metamodelling, according to Van Gigch, that real-world processes and models are compared, discussed and formulated (Van Gigch 1991, 133).

The following definitions are intended as a reference for the reader to consider the differences and similarities between the relevant urban concepts:

### Urban resilience

Urban resilience can be understood as the capacity of a city’s system, businesses, institutions, communities and individuals to survive, adapt, and grow.^5^ The term refers to the ability of an urban system, which includes constituent socioecological and sociotechnical networks across temporal and spatial scales, to keep up or promptly return to its required functions in the face of a disturbance, to agree to change and to quickly transform systems that limit its current or future adaptive capacity (Meerow, Newell, and Stults 2015, 39).

### HBUR

With a stronger focus on postregeneration preventive strategies, Wirilander (2021, 21f) described the concept of heritage recovery as the first aid that follows the disaster response process. Its objective is to stabilise the condition of heritage and to minimise or prevent the secondary damage that can sometimes be caused by disaster response. In line with this, Wirilander (2021, 202) went on to emphasise the importance of using qualified heritage professionals in collection recovery. According to Hallegatte (2018), in order to be resilient, the main principle of recovery is to ensure that the situation does not return to normal but rather improves after the process of rehabilitation. The ‘build back better’ approach is based on this principle and, in the case of heritage, includes dealing with integrity and authenticity values (Hallegatte 2018) and bringing heritage recovery close to the concept of urban development. For the purpose of this paper, we refer to urban recovery processes that focus on heritage and use it as a resource for heritage-based urban recovery.

### Urban regeneration

Urban regeneration is an outcome of the interplay between many sources of influence and the social, environmental, and economic pressures that force cities to adapt constantly (Roberts 2008). As described by (Roberts 2008, 9), urban regeneration also meets the opportunities and challenges that are presented in a particular place at a specific moment in time.

Rehabilitation strategies in cultural heritage sites should also include social aspects and ensure that repairing historic physical structures is part of an integral recovery plan that considers the intangible cultural heritage associated with the community (García 2019). The restoration process in cities has become more complex as it has become more extensive, which is why it is important to continuously assess the damage caused to urban cultural heritage while hostilities are still in progress. This assessment should be comprehensive and include history, archaeology, art, economics, sociology, population, and infrastructure such as roads, water, and sewerage systems. (Belal and Shcherbina 2021, 2). Additionally, it should take a step back from focusing solely on the material aspects of heritage (Smith 2006).

### Urban development

Urban development is a multidisciplinary research field focused on the improvement of urban areas by building. According to the EU, ‘Urban development covers infrastructure for education, health, justice, solid waste, markets, street pavements and cultural heritage protection. These constructions usually form part of specific sector programmes, including capacity building measures. (...) Rehabilitation and reconstruction comprises in particular social infrastructure following natural disasters or conflicts’ (European Commission 2021).

### HBUD

In this paper, we use the definition of HBUD developed by the city of Regensburg. It emphasises HBUD as urban development activities that actually have urban heritage as their starting point. Existing and specific urban heritage is analysed and valued at the beginning of the urban development process as a resource rather than an obstacle (City of Regensburg, Mühlmann, and Ripp 2012; Ripp, Hauer, and Cavdar 2019).

As illustrated in Table 1, HBUR and HBUD share many parameters; however, there is a significant difference in the level of urgency, which is naturally high in urban recovery, especially when responding to sudden disasters or crises. The time frame of urban recovery is usually more limited than that of ordinary urban development projects, although many urban development projects start with some type of crisis (such as climate change adaptation^6^ or as a response to social inequalities^7^). Although urban development and urban recovery are not the same, the two processes have more in common than not, for example, in terms of the high level of complexity that they share and the usefulness of a systemic approach when addressing them. They share a large variety of different stakeholders who are affected and could or should be involved. Both include built heritage, values, and functions and a ‘desired’ change, in contrast to urban transformation (Gustafsson and Ripp 2022).

**Table 1:** Comparison of approaches to heritage-based urban development, resilience, urban regeneration, and heritage-based urban recovery

Concept	Heritage-based urban development	Resilience	Urban regeneration	Heritage-based urban recovery
**Objective**	Improvement of quality of life	Stimulate adaptability	Area-based improvement of quality of life	Return to the quality of life before the disaster/crisis
**Key parameters**	Positivistic perspective	Enable systems to deal with changes/challenges	Improve the situation, usually in a defined area based on the identification of challenges/problems	Improve the situation to the original state and ‘build back better’
**Conceptual context**	SDGs/sustainable development paradigm	Models of resilience (e.g., from SHELTER^b^, Fig. 1)	Redevelopment to address urban decay, social policy, integrative strategies, improvement of specific areas, adaptive reuse	Integrated conservation, heritage valorisation, strategic development, disaster risk management, damage assessment, sustainable development
**Complexity of the system**	High	High	Fair (due to limited area)	High
**Level of (desired) cross-sectoral integration**	High	High	Fair (due to limited area)	High
**Time scale**	Long-term	Infinite	Defined time frame often determined by funding programme	Defined time frame
**Starting point**	Always possible	Continuous	Project-related	After crisis/disaster
**Urgency**	Low	Medium	Medium (based on identified challenges/problems)	High
**Context of international policies**	SDGs^a^, Urban Agenda^c^	Habitat III: The New Urban Agenda^d^, The Sendai Framework for Disaster Risk Reduction^e^ 2015-2030, The Hangzhou Declaration (2013)^f^, OECD Principles on Urban Policy^g^	New Leipzig Charter^h^, EU Cohesion Policy beyond 2020^i^	Habitat III^d^

^a^United Nations 2015a

^b^SHELTER 2020a

^c^European Commission 2016

^d^United Nations 2016

^e^UNDRR 2015

^f^UNESCO 2013

^g^OECD 2019

^h^European Commission 2020b

^i^European Commission 2020c

There is a wide range of literature on ensuring that cultural heritage is not harmed during disasters (Etinay, Egbu, and Murray 2018; Jigyasu 2016; O’Brien et al. 2015; Bianchi 2016), and there are extensive guidelines on how to treat cultural heritage during recovery. In contrast, the metamodel provides guidance on *how* to use cultural heritage for HBUR processes considering the system and the context. It is beyond the scope of this paper to analyse all existing models for these different concepts, but from a logical perspective, models are always restricted by their context, and transferability has limits that are rooted in these specific contexts. All four concepts have in common that they happen over a certain period of time and can be described as processes. The time frame of the processes varies (see Table 1) from a limited to an infinite perspective; at the same time, the starting point for such processes differs. While in HBUR, the initial point is a disaster or crisis, HBUD can begin anytime. Additionally, the scope of the objectives varies among the four concepts, from the improvement of a specific area (urban regeneration) to a broader possible improvement of the quality of life in HBUD processes.

In contrast to other ways of learning or gaining knowledge, such as the presentation of case studies (Pereira Roders and Bandarin 2019), the development of policy papers or recommendations such as the New Leipzig Charter (European Commission 2020a, b, c) or the proposition of specific strategies to be applied, as in the COMUS project (Council of Europe 2016), the metamodelling approach takes into account all relevant elements that are necessary for a ‘successful’ process.

Case studies present experiences from the real world, policy papers usually present visions (such as the green city in the Leipzig Charter), and strategies, while more transferable, are still often related strongly to a context. For successful HBUR processes, many elements must be taken into account. Great strategies and a perfect vision will not lead anywhere when, for example, there are no human or financial resources to implement them or the governance system is insufficient. The metamodelling approach is therefore one way to take into account all elements of successful processes.

The presented metamodel has the objective of presenting a tool to design and enhance the relevant processes and therefore goes beyond the transfer of ‘good strategies’, ‘good governance principles’ or ‘objectives’ by taking into account all relevant elements of these processes, including resources, processes and the key driver, who is the person mainly responsible for the implementation.

## The context for the metamodel: a new understanding of urban heritage and its potential to enhance the quality of life

To understand the applied method of metamodelling, it is important to consider the context from which and for which the metamodel was developed and where it can be beneficial. The general understanding of cultural heritage has changed considerably in recent decades. Sophia Labadi and William Logan maintained that today, several scholars see heritage as a social and political construct covering all places, artefacts and cultural expressions inherited from the past that, because they are seen to reflect and validate people’s identity as nations, communities, families and even individuals, are worthy of some form of respect and protection (Labadi and Logan 2015, xiii).

### The context of the metamodelling approach: a new understanding of urban heritage

The field of activities for the conservation of built heritage has grown from protecting individual monuments to more complex historic environments. Gregory Ashworth divided this development into three different paradigms that exist simultaneously: preservation (focused on authenticity), conservation (focused on adaptive reuse), and heritage (focused on meanings and experience) (Ashworth 2011). Dean Sully (2015) proposed similar ideas, highlighting the development of conservation practice from a focus on tangible heritage and preserving materials with the intention of as little intervention as possible to conservation based on people’s active participation, which instead prioritises human well-being. Joks Janssen and his colleagues studied how the preservation and conservation of built heritage in the Netherlands have changed in their relationship to spatial planning. They noted that three approaches have crystallised; conservation as a sector (silo thinking, where built heritage issues differ from spatial development), as a factor (built heritage is considered as a resource), and as a vector (built heritage is the starting point for sustainable spatial development). Although they have evolved individually, they are all equally relevant today (Janssen et al. 2017).

The importance of culture in a postindustrial economy in various contexts has recently attracted attention. Similar to the Italian cultural economist Pier Luigi Sacco’s theories of Culture 3.0 (Sacco, Ferilli, and Tavano Blessi 2018), we can study the development of conservation in three stages (Gustafsson 2019). Conservation 1.0 is about preserving and protecting a selection of culturally and historically valuable buildings through legislation and spatial planning. As in Sacco’s Culture 1.0, no clear economic value is generated in Conservation 1.0. The public sector is the main actor and often adopts a reactive approach that leaves initiatives to others. Conservation 2.0 is about maintaining and restoring the selected and protected buildings. In Conservation 2.0, the demand for built heritage increases, and economic values are created in a newly established market for historic buildings that consists of several different types of companies.

Finally, Conservation 3.0 is about adaptive reuse and starting to use historic buildings in ways that contribute to sustainable development. In Conservation 3.0, conservation is considered not a cost but rather an investment that is expected to lead to future returns in the form of social, environmental, and economic synergy effects for society as a whole. From this perspective, conservation can be understood as a contributor to sustainable development. It is important to point out that the stages are not about historical development, where society goes from one stage to another; rather, activities in the different stages occur simultaneously.

Several scholars have understood cultural heritage as a production factor and significant resource for the future and have suggested that investments in built heritage have impacts on inclusive, sustainable, and innovation-driven development. Conservation can be as a circular economy in which resources are reused in the sense of recycling instead of demolished buildings ending up as waste. After all, conservation is about taking care of existing resources, and houses that are not demolished can be said to be the most sustainable buildings.

### Urban heritage as a resourceful system

To embrace the potential of the proposed methodology, it is also necessary to consider the bigger picture and how cultural heritage can be understood as a system. For this paper, we focus on urban heritage as a system of tangible and intangible heritage, a well-developed theoretical discourse and a multidisciplinary platform that includes dimensions of use and functions that interact directly with communities and citizens. We follow the ideas of Ripp (2021), who stated that urban heritage can be the foundation for increasing the quality of life of these communities and users. In this sense, cultural heritage is much more than an object; it can be better described as systems and processes (that belong to and are recognised as such by the communities, groups or individuals that create, maintain, and transmit heritage) (Ripp 2018).

At the end of the 12th century and the beginning of the 21st century, a deeper understanding of cultural heritage as a system gradually started to evolve (Ripp and Rodwell 2015). This system comprises not only a collection of built monuments that are arranged and located in a specific way (ensemble) but also, for example, the users, values, functions, and ongoing processes of the monuments. The monuments themselves are subject to changes (such as changes in perception, use, or values) that are connected to the relevant communities and their activities (such as using, reusing, financing, destroying, photographing, and buying). The role of these communities is increasingly recognised in international framework conventions such as the Faro Convention (UNESCO 2005a, 2005b), where the fundamental role of civil society was valued; this concept was later also reflected in the Operational Guidelines for the Implementation of the World Heritage Convention (Ripp 2018, 2).

Cultural heritage as a system includes tangible and intangible heritage, people who are doing something with this heritage and those who are influenced by its values and processes. Consequently, a much broader view of cultural heritage is needed, and different disciplines must be involved in an inter- and cross-disciplinary way. This systemic understanding was emphasised, for example, by Fusco Girard (2013) and was also reflected in UNESCO’s 2011 Recommendation on Historic Urban Landscapes (UNESCO 2011), which ‘defines the broader urban context to include: the site’s topography, geomorphology, hydrology and natural features, its built environment, both historic and contemporary, its infrastructures above and below ground, its open spaces and gardens, its land-use patterns and spatial organisation, perceptions and visual relationships, as well as all other elements of the urban structure.’ This holistic and systemic understanding is in line with a systemic approach that can be found in integrated urban development and the UN sustainable development goals (SDGs), among which individual goals are connected to each other, with many being more transversal than sectoral. Bishop also supported this understanding and highlighted that heritage is a process that is shaped by and morphs with the needs of the present and can be manipulated to favour those in power (Bishop 2014, 199).

The combination of the urban resilience concept developed within the SHELTER project and the metamodel approach should be understood in the context of cultural heritage as a system; therefore, a systemic approach, based on a systemic view of the world, allows a better understanding of urban heritage. A systemic view of the world is determined by the perspective that in addition to linear relations between different entities, complex relations and interactions exist that do not always follow a linear logic but may change or react in a dynamic and complex way (Ripp 2021).

For the use of cultural heritage for postcrisis recovery, this systemic and holistic understanding of cultural heritage is essential, as crises usually affect civil societies at a range of levels that are connected and can hardly be addressed on the level of sectoral interventions (SHELTER 2020a).

The potential benefits of cultural heritage include economic, environmental, and social benefits and can be related to the quality of life concept of the World Health Organisation (WHO). The WHO outlined quality of life as an individual’s perception of his or her position in life in the context of the culture and value systems in which he or she lives and in relation to his or her goals, expectations, standards and concerns (World Health Organisation 2020). While the UN SDGs are based more on quantitative indicators and have been developed in the tradition of, for example, the Human Development Index, (various) quality of life concepts are more connected to qualitative factors of individual perceptions and well-being, an area that has often been connected to cultural heritage in scientific research and relevant policies. However, if we follow the Council of Europe’s cultural heritage definition that we have just elaborated, it becomes clear that dimensions such as ownership, reflection, and expression of values and beliefs are closely connected to the quality of life concept of the WHO, which is also based on the context of culture and value systems and, e.g., expectations. This social dimension of cultural heritage is also essential to the concepts of HBUD and HBUR that we address in this paper. It is not sufficient to focus on hardware, e.g., urban heritage buildings, because the social dimension is also significant important. The full picture can be considered through a systemic understanding of urban heritage. UNESCO’s Recommendation on the Historic Urban Landscape (UNESCO 2011) also uses quality of life as a point of reference.

Cultural heritage, especially if understood as a system and process (Ripp 2018), goes far beyond physical qualities and is embedded in the lives of many communities not only through the use of cultural heritage but also through sociological dimensions such as identification with heritage (Whitehead et al. 2021), a sense of feeling at home (Morley 2001; Duyvendak 2011), and values from the past that contribute to these feelings (Lipman 2018). The role of cultural heritage in physical health became apparent during the COVID-19 crisis, when large parts of urban populations rediscovered historic green areas as the only (accessible) resource for recreation, physical activities, and sport (Council of Europe 2020; Euro Cities 2020). Engagement with heritage through community-based projects that can address social isolation and enhance people’s quality of life has been demonstrated to improve social and mental well-being. Health-enabling spaces are thus concerned with complex interactions that can be physical, mental, emotional, spiritual, societal, and environmental (Williams 2007; Power and Smyth 2016). Taking this into account, cultural heritage in postcrisis recovery (using the definition of resilience from the SHELTER project and focusing on the recovery/build back better phase) can be understood as a resource for sustainable development (Petti, Trillo, and Makore 2020; Nocca 2017; Fairclough et al. 2008; Murzyn-Kupisz and Jarosław Działek 2013; Jeon and Kang 2014) and can contribute to quality of life in times of crisis. In contrast to earlier thinking that conservation of built heritage is a cost to society and an obstacle for urban development, a number of recent projects have been implemented in which preservation and conservation of cultural heritage have been understood as investments with obvious social, environmental, and economic returns (Brandt-Grau et al. 2015; Cultural Heritage counts for Europe 2015; Stanojev and Gustafsson 2021; Foster and Saleh 2021; Roszczyńska-Kurasińska et al. 2021). Thus, cultural heritage, and especially historic urban and rural landscapes, have been used in systematic and structured processes to achieve general objectives of sustainable development at local and regional levels. Therefore, how we use cultural heritage is of great importance.

The strategic use of built heritage as a starting point for urban development can be understood as an innovation in itself because urban development processes often have a different departure point, and cultural heritage representatives are introduced to the process only at a later stage (Ripp 2021). Beyond this, cultural heritage in general and historic urban and rural landscapes, in particular, has been increasingly connected with innovation systems. There are opportunities to use cultural heritage in social innovation integration, where pluralism of values, active community participation, and social organisation models can stimulate collective cocreation, enabling social access and cohesion. (Sacco, Ferilli, and Tavano Blessi 2018). Cultural heritage can underpin social cohesion by supporting interaction among various sectors, industries or systems (Buscema et al. 2019; Gustafsson and Lazzaro 2021), and adaptive reuse of historic buildings can become an important aspect of heritage strategies in connection with a circular economy (Gravagnoulo and Fusco Girard 2017; Janssen et al. 2017; Fusco Girard and Noccia 2019; Gustafsson 2019; Stanojev and Gustafsson 2021).

### Using heritage for urban development

There are various published case studies on heritage and sustainable development (Hassler, Algreen-Ussing, and Kohler 2002; Loulanski and Loulanski 2016; Peou et al. 2016) with a project-level focus, and a number of meta-studies have identified the potential benefits of cultural heritage. A metastudy by the University of Krakow and several partners was published in the European Commission-funded project Heritage Counts for Europe. The results clearly show the role of cultural heritage in enhancing the attractiveness of cities and regions in Europe, stimulating (private) investment, skills training, and (start-up) business (Cultural Heritage Counts for Europe 2015, 19). The identified benefits are numerous and range from soft effects, such as creating narratives for better city marketing and providing sources for innovation and possibilities for lifelong learning, to quantitative effects, such as good investment returns, tax revenues, and the creation of jobs (Cultural Heritage Counts for Europe 2015). Other authors, such as Engelbert Ruoss (2016), Donovan Rypkema (2005), and Terje Nypan (Nypan and Warr 2015), referred to economic benefits and the potential for job creation.

Cultural heritage has been regarded as a resource, and conservation of built heritage has been regarded as a production factor. Several studies have shown that cultural heritage has a positive effect on nations’ GDP and on cities’ and regions’ competitive advantage in relation to the rest of the world (Dümcke and Gnedovsky 2013). Cultural heritage is also recognised in relation to innovation, growth, competitiveness, and welfare, as it is understood as a production factor in an economic context and in policy documents (Stanojev and Gustafsson 2021; Gustafsson and Lazzaro 2021). Tourism is an important source of income; however, only a part of the positive economic contribution comes from cultural heritage. Conservation, renovation, and maintenance represent more than 25% of the value of the construction industry in Europe (Nypan and Warr 2015). Historic areas also attract skilled professionals to live and work, which implies that cultural heritage can be a platform for innovation and improve the long-term competitive advantage of cities and regions (Valentina et al. 2015).

Heritage-led urban development has been a topic of several recent research projects (e.g., Cultural Heritage Counts for Europe 2015; Ferilli, Gustafsson, and Sacco 2017; Xie and Health 2018; Kee and Chau 2020). In Europe, the EU Horizon 2020 research and innovation framework programme has financed several projects on this subject. For example, the objective of the Circular models Leveraging Investments in Cultural heritage adaptive reuse (CLIC) project was to apply circular economy principles to cultural heritage adaptive reuse to achieve environmentally, socially, culturally, and economically sustainable urban/territorial development with a focus on adaptive reuse (http://clicproject.eu). The Regeneration and Optimisation of Cultural heritage in creative and Knowledge cities (ROCK) project focused on historic cities as laboratories to demonstrate how cultural heritage can be a powerful engine of regeneration, sustainable development, and economic growth for urban areas (https://rockproject.eu). In the Heritage for Rural Generation (RURITAGE) project, the focus was not merely on urban development but on enhancing local heritage sustainably for regional and community development. This project aimed to regenerate areas with the help of the Systemic Innovation Areas (SIA) framework, which identifies unique heritage potential within rural communities (https://www.ruritage.eu).

In parallel to the scientific findings, the various international organisations that are active in the field of cultural heritage have developed a common understanding that heritage is potentially a powerful resource for urban development (European Commission 2014, 2015; UNESCO 2016; World Bank 2021). The New Urban Agenda (NUA), which was adopted in 2016 at the United Nations’ Conference on Housing and Sustainable Urban Development (Habitat III) in Quito, represents a shared vision and a road map for how cities can serve as engines of prosperity and centres of cultural and social well-being while protecting the environment. The NUA establishes a comprehensive and progressive role for cultural heritage in urban development and expresses the role of cultural heritage in resilience and as a driver of social mobility, equity, and inclusive economic development in the urban economy. The UNESCO report Culture: Urban Future (2016), which was launched at the UN Habitat III Conference 2016 in Quito, explores the role of culture in sustainable urban development from three perspectives: building on the power of culture to promote human and inclusive cities, improving the quality of built and natural environments through culture, and integrating culture into urban policies to foster sustainable urban development.

### From HBUD to HBUR

The strategic use of cultural heritage for recovery in a postwar setting was examined by Katherine Louise Bishop (2014), who asserted that heritage has a significant role in both conflict and recovery from conflict; however, its role in the postconflict landscape has not been fully explored conceptually and, of interest for this paper, its role has not been fully implemented in recovery processes by practitioners (Bishop 2014, 2). In her dissertation, Bishop developed an integrated heritage assessment framework (IHAF) and tested it on the case of Dubrovnik. One of her key findings was that heritage has a continuing and considerable impact on the way that societies recover from conflict (Bishop 2014, 198). She offered a compelling argument and motivation to follow this path by exploring how the metamodel for HBUD can be transferred to the HBUR scenario. Bishop further elaborated that if we understand heritage as a process in postwar scenarios, heritage changes due to specific meanings and values that may be at the core of conflicts and, for example, are often attributed to buildings. This finding further emphasises the case for understanding urban heritage as a system and process (Ripp 2018). Zachary Jones examined how initiatives such as the European Capitals of Culture can contribute to urban development and urban recovery, and it is noteworthy that urban heritage served as a resource and setting for events and projects that then contributed to urban development and recovery in the cases that he examined (Jones 2020).

## Methodological approach: Metamodelling

In response to the research question, an abstract metamodel was developed that can be used in different scenarios regardless of the specific environment. While models are strongly determined by their specific environment, metamodels are on a more abstract level and are usable in a more universal way (Van Gigch 1991). They are also better able to address the systemic nature of the underlying logic of development. With a metamodel, it is possible to obtain a deeper understanding of the relevant factors and processes. With this broadened perspective, the interrelations within a system and the associated challenges can be better addressed (Ripp 2021). This is why the metamodel can also be used for HBUR, which shares many qualities with urban development (see Table 1).

For the planning of HBUR processes, and especially the scoping phase (=preparatory phase) of such processes, a metamodel in which the phases and elements of heritage-based development (HBD) processes are described can be used to design and structure HBUR processes for a specific urban setting with a specific historic urban fabric. For this purpose, a model of one successful HBD process is not appropriate because each model describes methodologies and tools that are relevant in specific cases with specific preconditions (in developed countries, for example, with a wide range of civil society and nongovernmental organisations, highly diversified local administrations, and access to financial resources and experts). Therefore, if a specific HBD model, such as the management plan approach (Scheffler, Ripp, and Bühler 2009) or the Halland model based on the trading zone concept (Gustafsson 2009), is used to scope local HBD processes in a divergent setting, where all parameters and preconditions are different, the model may soon prove to be too limited and will likely face various challenges – as was experienced in an attempt to transfer the HerO model to the COMUS project (Council of Europe 2016; Ripp 2021).

The metamodel proposed in this paper was developed using three noteworthy projects as cases in a mixed-methods research approach. Projects that had successfully used cultural heritage as a starting point for urban development were selected. Further selection criteria included the explicit use of built cultural heritage for urban development and the implementation of an integrated approach that brought together different uses and stakeholders, indicating a systemic understanding of the field, in contrast to a traditional preservation-centred narrative, in which the safeguarding of cultural heritage is the first and final objective. The selected cases all demonstrated new narratives and the use of cultural heritage as a tool to achieve other objectives (Ripp 2021, 53). The cases were analysed using grounded theory; thus, it was possible to develop abstract elements through design research methodology that served as the starting point in constructing the metamodel. Logically, the nature of the selected case models influences the universality of the metamodel. The integration of case models from different cultural spheres would have made the metamodel even more universal.

However, the selected case models are relevant for the following reasons:

1. The case models (HerO and the Halland model) were developed in what can be described as a postcrisis context (City of Regensburg, Ripp, and Scheffler 2011; Gustafsson 2009). When the HerO project started, Europe was shaken by the financial crisis that had started with the crash of the Lehman Brothers Bank. The effects of the financial crisis were severe and visible throughout Europe, and the HerO project responded with the structured and strategic use of cultural heritage for urban development. The starting point of the Halland model was a labour market and economic crisis in Sweden in the early 1990s. The market for the construction industry collapsed, resulting in a high rate of unemployment. The cultural heritage sector took the initiative of establishing cross-sectoral cooperation to recover the regional economy, save and create jobs, and preserve traditional building techniques and historic buildings by offering unemployed construction workers jobs in conservation projects. The COMUS project was established in a governance context that can be understood as a political crisis, with the countries of the eastern partnership in a somewhat unstable situation, not exactly knowing whether they were following more European values and coming closer to the European Union or keeping or returning to a closer liaison with the former USSR (Ripp and Stein 2018).
2. While we can argue that urban transformation is always happening, urban development can be described as urban transformation that is guided by specific objectives. This framed urban transformation is similar to the urban recovery process, as we have already elaborated. Therefore, the success factors for HBUD are similar to the success factors for HBUR processes. ‘Successful’ HBUD projects were analysed to develop the metamodel, and the ‘successful’ elements included in the metamodel can also be applied to the postrecovery scenario. The following three case models were used:
  1. The URBACT II HerO project involved nine European cities using heritage management plans as a tool for urban development (City of Regensburg, Ripp, and Scheffler 2011);
  2. The Halland model project transformed the mechanisms of the regional labour market in concert with cultural heritage into training opportunities for unemployed construction workers and developed planning mechanisms for the adaptive reuse of historic buildings, which then stimulated regional sustainable development based on cultural heritage (Gustafsson 2009); and
  3. The COMUS project, in partnership with the Council of Europe, European Commission, and Organisation of World Heritage Cities, used several medium-sized and small Eastern European towns with strong local community involvement in cultural heritage as a starting point for urban development (Ripp and Stein 2018).

In parallel, but in a different context, the SHELTER project, funded by EU HORIZON 2020, was also used for this research. ‘The overall objective of SHELTER is to establish a cross-scale, multidimensional, data-driven and community-based operational knowledge framework for heritage-led and conservation-friendly resilience enhancement and sustainable reconstruction of Historic Areas to cope with Climate Change and natural hazards’ (SHELTER 2021). This project differs from those previously mentioned in that its scope includes sudden disasters as well as more slowly occurring impacts of climate change. The conceptual model of resilience (Fig. 1) is based on the SHELTER project.

## A (new) role for cultural heritage in resilience and postcrisis recovery

People’s individual and shared interpretations and experiences or understandings of heritage are crucial (Ripp and Hauer 2017). Notably, these interpretations are constantly changing. Nothing is heritage in itself unless it is perceived and used as such. Cultural heritage can be regarded as ‘the only legacy that cannot be inherited; instead, it must constantly be acquired’ (Kulturdepartementet 2017, 63, authors’ translation).

The United Nations’ 17 global goals for sustainable development were the first such document to mention the protection of cultural heritage (United Nations 2015a, b). Even though cultural heritage is mentioned only in Target 11.4 with the aim of strengthening efforts to protect and safeguard the world’s cultural and natural heritage, it plays a vital role in achieving most of the goals, for example, as an enabler of social cohesion and inclusion and as a driver of equity and inclusive economic development. Additionally, in historic environments, it can improve the liveability, resilience and sustainability of both older and newer areas.

The starting point of the NUA is that urbanisation can be a powerful tool for a sustainable future and highlights the role played by tangible and intangible heritage in strengthening social participation and exercising citizenship. The agenda calls on interested parties to support leveraging cultural heritage for sustainable urban development and to recognise its role in stimulating participation and responsibility (UNESCO 2017). In the UNESCO Recommendation on Historic Urban Landscape, cultural heritage is also linked to creativity and development (UNESCO 2013).

The theoretical framework of resilience as it is used in the SHELTER project is aimed mostly at postdisaster scenarios. However, the line between sudden disasters and slowly occurring crises is anything but distinct. The SHELTER project focuses on examples of disasters and impacts on cultural heritage caused by climate change, many of which are not occurring suddenly. The often artificial distinction between disasters and crises is noted but will not guide the scope of this research to focus exclusively on only one of the two. The resilience qualities that cities need to respond to disasters and crises are similar to a certain extent, as they require a (more or less rapid) cross-sectoral and integrated strategic response; however, this response is defined in a specific cultural context of administration and differs depending on the nature of the disaster or crisis. In this paper, we focus on the commonalities that can be incorporated into a structured planning process. Of course, when the metamodel is applied to a specific context and a specific setting determined by a specific disaster crisis, the real-world entities are very different. To address this problem, this paper employs a literature review and the transfer of the metamodel for HBUD as recently developed in a dissertation by Matthias Ripp, followed by a discussion in which a scenario for the use of the metamodel for postcrisis recovery is developed.

The term ‘resilience’ is used in various ways, and we adopted a recent concept from the EU Horizon 2020 SHELTER project. We wanted to be precise about the phase of resilience in which the metamodel can be used, and the SHELTER project concept of resilience suited this purpose, as it considers different phases before, during, and after a crisis. The four defined phases presented in the project are relevant for HBUR. Risk prevention and development and enhancement of risk preparedness occur mainly before an actual crisis has started to have an impact. Conversely, the response phase starts immediately after a crisis or disaster, which leads to the subsequent recovery/build back better phase. The difference between disasters and crises can be defined mainly on the basis of their temporal dimensions and degree of suddenness. While urban heritage can contribute significantly to prevention and preparedness (Ripp and Lukat 2017), this paper focuses on the response and recovery/build back better phases. The presented model is contextualised in three sequential disaster phases: the predisaster phase before the crisis, the transdisaster phase during the disaster, and the postdisaster phase. However, as illustrated by the (dotted) visualisation of these three (linear) phases of disasters (or crises) in Fig. 1, they are not to be understood from a binary perspective, and the boundaries between the phases are open and fluid. The assessment and monitoring in Fig. 1 also show the types of tools used in each phase. We propose an additional tool in this article, the metamodel for HBUD (Ripp 2021), which can be used mainly in the postdisaster phase.

On a global scale, cultural heritage is frequently affected by disasters such as earthquakes, floods, fires, and crises, which can be short or long term, for example, climate change and economic and health crises (Spennemann and Graham 2007; City of Regensburg, Ripp, and Bühler 2009; SHELTER 2020a, b). The aims and objectives of the responses to these crises vary according to their specific nature. The SHELTER concept of resilience is structured in three phases: prevention, recovery, and build back better. In each phase, different processes are relevant to enhancing resilience. The model considers the different contexts and needs in each resilience phase, which can help to enhance the understanding of which skills, expertise, decisions, and resources are necessary for each phase (they are not necessarily the same in each phase). Building on these three phases (as outlined in Fig. 1), the objectives and potential roles of cultural heritage can be described according to the separate phases (Table 2).

**Table 2:** Potential role of cultural heritage in the SHELTER concept of resilience. Based on SHELTER 2020 and our own considerations

SHELTER concept phase of resilience	Potential objectives	Potential role of cultural heritage
Prevention	Avoid disaster and crisis	Context/element of the scoping phase
Preparedness	Enhance preparation for potential disaster and crisis	Asset to be protected
Response	Emergency reactions	Resource
Recovery/build back better	Increase the quality of life for local communities	Resource

Table 2 illustrates the role of cultural heritage in the four resilience phases of the SHELTER project (see Fig. 1). Based on conceptual resilience frameworks, the project further developed the potential roles of cultural heritage, which changed throughout the different resilience phases. To understand the proposed application of the metamodel for HBUD (Ripp 2021) in a postdisaster scenario, it is useful to consider the roles of cultural heritage in relation to resilience. This paper evaluates whether the metamodel can be used in a postcrisis scenario and explores the limitations of the metamodel through the roles of cultural heritage in the different resilience phases; this research was not intended to enhance risk preparedness. These changing roles reflect the underlying systemic logic that is apparent in the SHELTER concept of resilience and in the metamodel.

## Method: a metamodel for HBUD

There is a range of best practice examples and role models from specific environments; however, most of them cannot be applied and replicated everywhere. Attempts to transfer successful examples from a specific context to another context often fail. One challenge is that there is no universal method to make heritage investments work as a catalyst for urban development because a model is not capable of being universal. A possible solution to this challenge is developing a metamodel that is based on successful models from different contexts and can finally be applied universally. A metamodel is on a higher level of abstraction than a model and is therefore independent of the context of a specific model. Metamodels, following the theory of John P. Van Gigch, are systemic by nature and usually represent systems (Van Gigch 1991, 121ff). As elaborated above, cultural heritage is increasingly understood as a system; therefore, a metamodel is suitable to represent HBUD and recovery on a conceptual level.

**Fig. 2:**
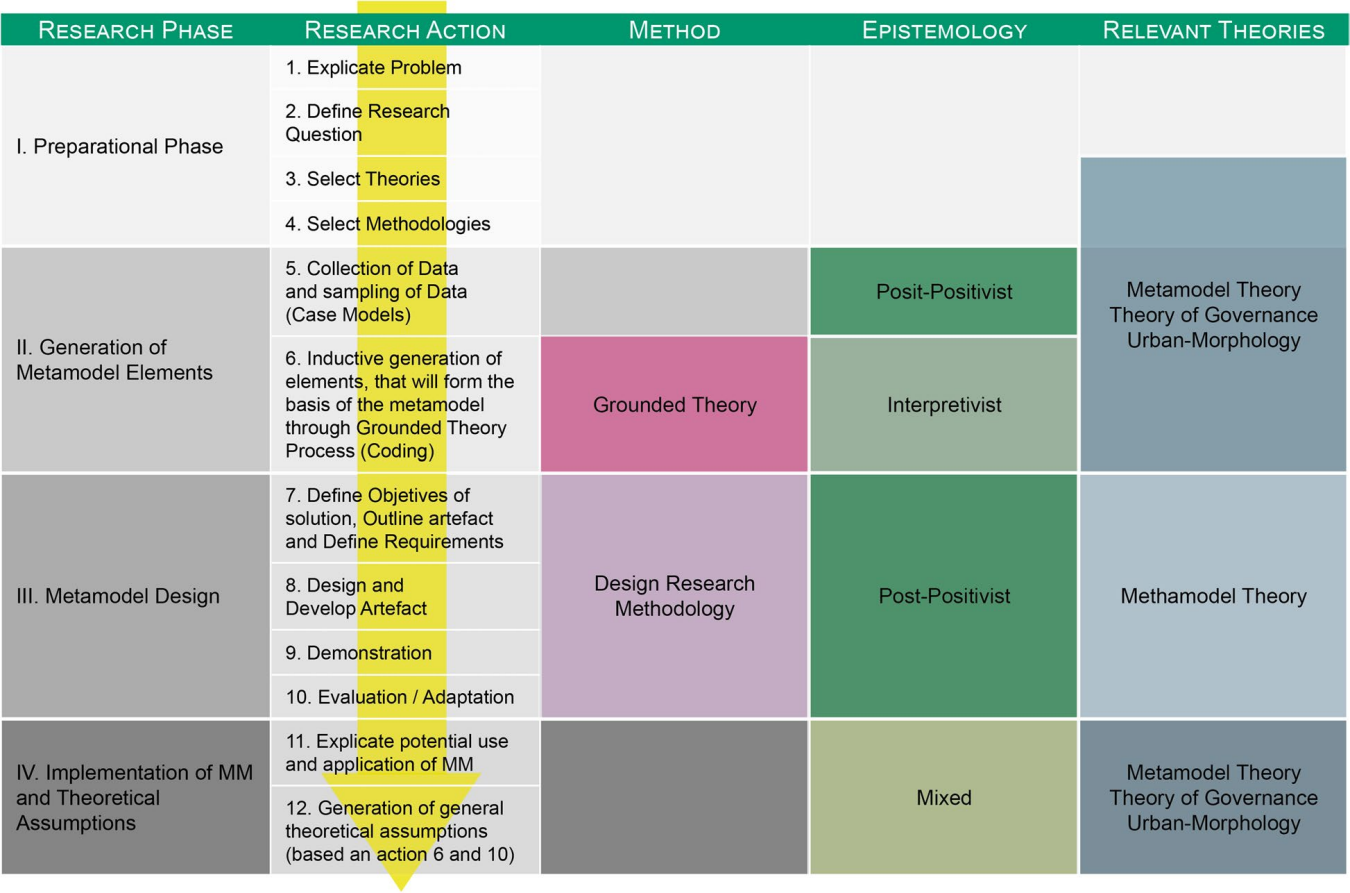
Mixed-methods research design for the development of the metamodel (Source: Ripp 2021)

A sample of case models, namely, the HerO project, COMUS project, and Halland model (City of Regensburg, Ripp, and Scheffler 2011; Ripp and Stein 2015; Gustafsson 2009), were selected according to the criteria developed by Ripp (2021, 53). They were first analysed using grounded theory to develop a set of abstract elements for the metamodel. It is beyond the scope of this paper to explain the entire process of how the metamodel was developed. However, a general overview of the research design can be found in Fig. 2, which demonstrates how grounded theory and design research methodology were combined in a mixed-methods research approach.

Figure 2 shows how the abstract elements of the metamodel (entity groups and domains) were developed from the three case models using grounded theory. In this process, the level of abstraction increased.

Clear comprehension of the difference between models and metamodels is of the utmost importance for understanding the objectives of the metamodel. According to John P. Van Gigch, the (systemic) world can be seen on three distinct levels:

A. The real world – where problems and solutions are taking place.
B. The level of modelling – models are, e.g., designed to transfer a working solution from one context to another; they show a medium level of abstraction and are still deeply rooted in the cultural context from which they are built.
C. The level of metamodelling – metamodels are logically one step above models and consist of elements with a great level of abstraction. They are used, for example, to develop, select or evaluate models. They are applied not directly but universally (see the following section on the Universality of the Metamodel and the definitions of model and metamodel in the introductory section).

As indicated by the definitions, models used in connection with cultural heritage and urban development, for example, the SCENE model, are not rare (Grosskurth and Rotmans 2005). Metamodelling has significant potential for HBUD, and the benefits of this approach are as follows:

A. The metamodel can better represent the general systemic nature of HBUD and its processes.
B. The metamodel is built of abstract elements (herein referred to as entity groups on one level of abstraction and domains on the level above) and can therefore be transferred to any environment (where it must be filled in with the specific context).
C. The metamodel represents all abstract elements of successful HBUD projects and therefore has a much broader base than a normal model.
D. The metamodel can be used to choose, design, evaluate, and improve existing models for HBUD.^8^

In brief, metamodels are used differently than models. They should never be compared to each other without fully considering their logical and epistemological differences (Ripp 2021).

**Fig. 3:**
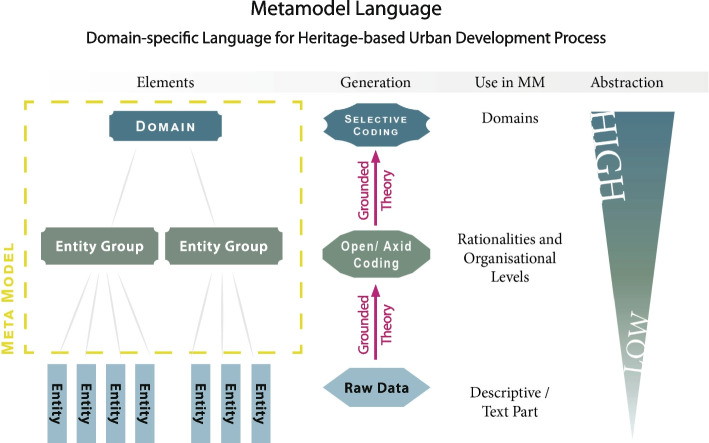
Development of the elements of the metamodel (Source: Matthias Ripp)

The development of the elements of the metamodel (Fig. 3) based on three successful case models from different environments ensures the best possible representation of successful processes. While a full elaboration of the mixed-methods research design of the metamodel is beyond the scope of this paper, basically, a postpositivist epistemology was used to select three case models that were successful, in the sense that they all reached the objectives that were defined in the beginning. These three case models were then examined (through their final reports) using grounded theory. Every line of text was rigorously analysed through open, axial, and selective coding to extract the elements of the metamodel (Fig. 3). The epistemology in this analysis was interpretative because, as Glaser (1976, 1978) described with the term ‘theoretical sensitivity’, the grounded theory research process involves strong elements of individual cultural context and worldview.

In the second step, using a postpositivist epistemology design, the research methodology was applied to define the requirements for the metamodel. Considering these requirements, the metamodel was built from the metamodel elements, and a set of scenarios in which the metamodel can be applied was developed. It can be used, for example, to design, improve, and evaluate new and ongoing HBUD processes and for various other purposes, including developing curricula and capacity building.

According to Easterday, Rees Lewis, and Gerber (2018), design research methodology can be understood as a series of structured steps towards the generation of an artefact. The different steps used in this research were based mainly on Peffers et al. (2007) and Niedderer (2009) and consisted of the explanation of the problem, the definition of objectives for the solution, the outline of the artefact, and the definition of requirements. Then, the artefact was designed and developed (based on the elements that had already been elaborated through grounded theory from the three case models), demonstrated (through simulation using the city of Regensburg as an example), evaluated, and finally communicated.

**Fig. 4:**
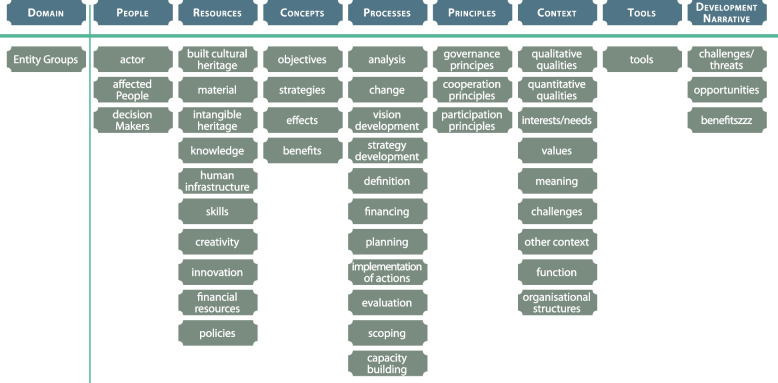
Elements of the metamodel, generated with grounded theory (Source: Matthias Ripp)

**Fig. 5:**
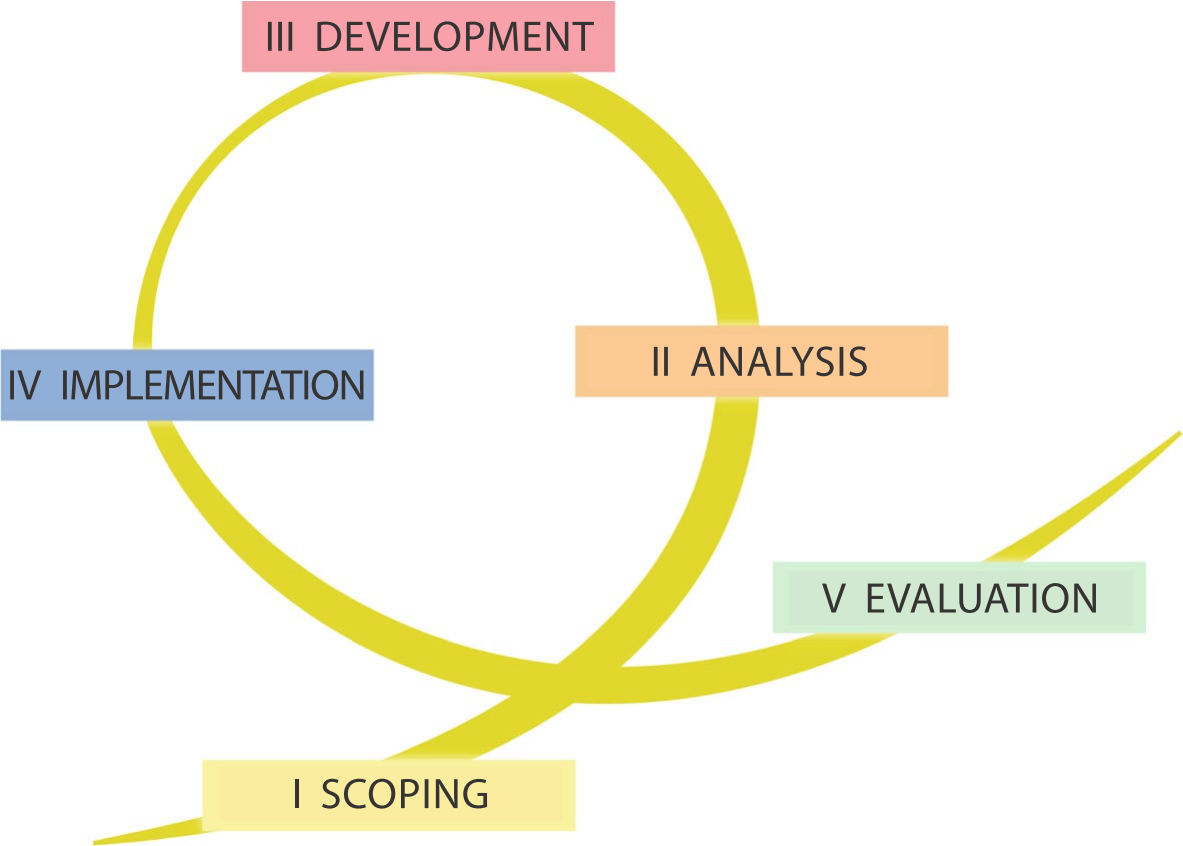
The five phases of heritage-based urban development (Source: Ripp 2021)

The metamodel can ensure, for example, that all relevant elements (see Figs. 4 and 5) are taken into account when such processes are prepared. The five phases of the metamodel can help us understand that scoping before starting thorough preparation is essential for successful projects. The metamodel shows the complexity and relationships of different entities and domains that are relevant in HBUD processes and is also a practical tool that can be applied universally in any case where heritage is used for urban development, for example, to prepare, evaluate, or improve HBUD and HBUR processes.

## Universality of the Metamodel

As previously mentioned, the difference between a metamodel and a model must be emphasised. A metamodel is on a much higher level of abstraction than a model and cannot be used directly. By nature, any metamodel, using John P. Van Gigch’s metamodelling theory, is made up of abstract representations of reality. Metamodels can be used to make or select models but cannot be applied directly. Consequently, the critique that a metamodel from a specific environment cannot be applied in a different one does not consider the nature of metamodels. In contrast to models, no metamodel is connected to a specific environment. Metamodels can be constructed from examples that are always connected to specific environments, but they naturally demonstrate a more universal view through the process of abstraction involved in metamodelling and the resulting abstract representations. Ultimately, this is exactly why we use a metamodel – to obtain an abstract universal view of something – and not a model, which is strongly determined by its context.

However, it is still possible to reach the limits of any metamodel if it is filled in with a specific context and a specific entity can be found in its abstract representations. In the case of the metamodel at hand, this remains to be proven.

The metamodel is built of elements from two distinct levels of abstraction. Domains are on the highest level of abstraction, and ‘people’ is one example of such a domain. Entity groups are on the lower level of abstraction and are more specific but still abstract; for example, in the domain ‘people’, one entity group is ‘decision makers’.

**Fig. 6:**
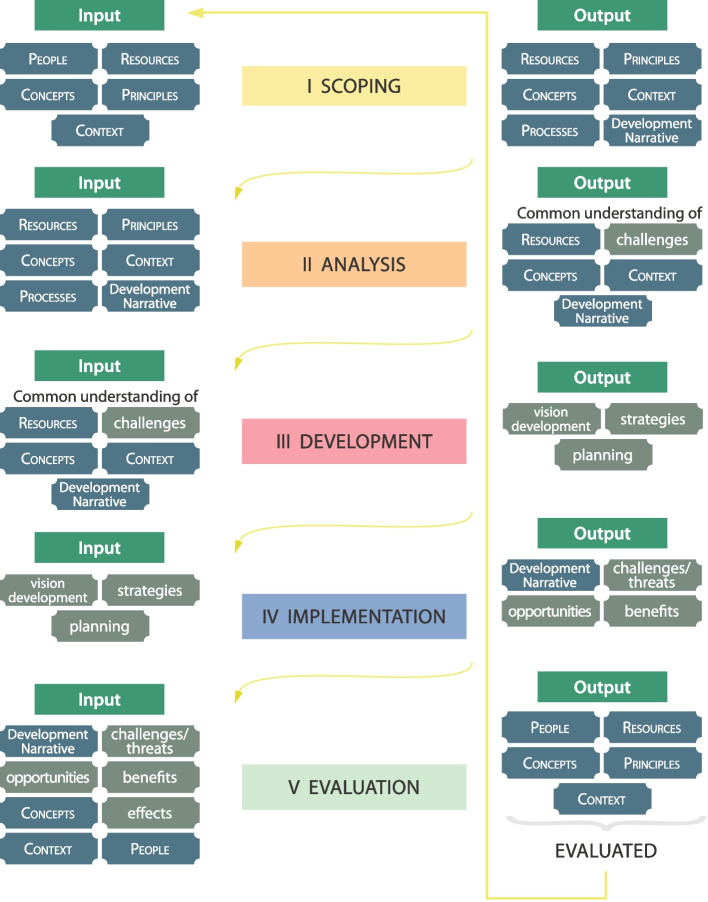
Logic connections between the five phases of heritage-based urban development (Source: Ripp 2021)

The overall structure of the metamodel can be represented as a spiral (Fig. 5) that consists of five phases: scoping, analysis, development, implementation, and evaluation (Ripp 2021). For a more detailed description of each of the five phases, including relevant domains and entity groups for the specific phases as well as inputs and outputs, please see Fig. 6.

**Fig. 7:**
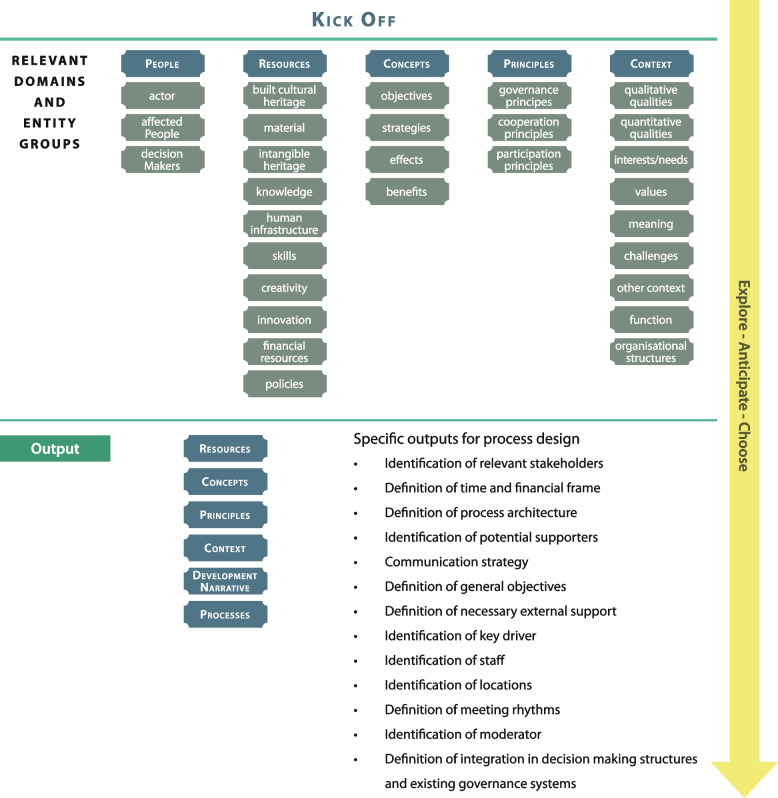
The scoping phase of heritage-based urban development is used to prepare the entire process (Source: Ripp 2021)

The five phases are logically connected and built on each other; however, the consecutive order is more an ideal representation than a prescribed path that must be strictly followed in the real world. Different domains and entities (representing subjects, objects, and processes) are relevant in each of the five phases, and the result of one phase generally provides the starting point (input) for the following phase.

For example, the first phase, the scoping phase, represents the preparation of HBUD projects (Fig. 7):

The scoping phase is of extraordinary importance because it lays the groundwork for the entire process and defines the necessary skills and roles.

Because of the broad basis of the case models (HerO project, COMUS project and Halland model) from which the metamodel was developed, the abstract nature of the metamodel can in principle be applied in any setting. Of course, the application is not direct, but the metamodel can be used in specific cases for which it must be translated or filled in with specific entities from the real world, which are not present in the metamodel. For example, the abstract representation people (domain) and decision makers (entity group) in the case of a German city could be filled with the specific real-world entity (e.g., the City Council of Regensburg – cultural committee). However, the use of the metamodel can ensure that the process is structured accordingly and that all different phases, processes, and elements are included based on successful HBUD projects. The metamodel represents the HBD process on an abstract level and can be used to select appropriate models and methods to translate the abstract metamodel, for example, to a specific road map that can be implemented in a specific context (Ripp 2021). However, the metamodel can never be applied directly. It must always be related to a specific context, which is a strength in one way and a limitation in another.

## Analysis: the use of the model for recovery after crisis: the scenario ‘use urban heritage for recovery/build back better’

The metamodel can also be used in the recovery/build back better phase, as described in the resilience model of the SHELTER project (SHELTER 2020a, b). The users of the metamodel, in this case, could be officers from the public sector at the local, regional, or national level. It can also be used by external experts who are contracted to design recovery processes that are based on urban cultural heritage. The metamodel could be used to design a heritage-based recovery process for a specific case.

The metamodel can be beneficial in the preparation and planning of such processes. One result of the analysis of the three case models is that the preparation of such processes is most important for their success (Ripp 2021). In the scoping (preparation) of HBUR processes, the metamodel can be used to understand the separate phases of an HBUR process and to develop a realistic time scale for each phase and the whole process. It can help to identify what entities are relevant on the local level in the HBUR process (based on the domains and entities that are represented in the metamodel) and to define the role of the key driver in the process. In cases where the necessary skills to facilitate such processes are not available in the local team, the metamodel can highlight the need for external expertise. The governance of the process and how decision makers should be integrated can also be understood with the help of the five phases of the metamodel, and the necessary financial resources can be recognised and defined. The metamodel can also help to generate general objectives for local HBUR processes and to determine the role of stakeholders and develop initial ideas for selecting them and integrating them into the process (Ripp 2021).

One advantage of using the metamodel to design HBUR processes over other methods, such as a spontaneous approach or following a specific case or model, is that all potentially relevant entity groups and domains are respected, and the different relevant phases that are part of the HBUR process are understood and integrated into the project and process architecture. Due to its abstract nature, the metamodel can be applied with specific entities that are not relevant or present in other cases, which is a further advantage. Consequently, it is more universal than the transfer or application of any model. The metamodel also emphasises the importance of the scoping phase, which has been identified as crucial for a successful HBD process and enables the persons responsible for the design of the HBUR process to start with a deep and wide understanding of the nature of the process, including potential pitfalls and challenges.

The following list of concrete steps is an example of how the metamodel can be used in the scenario ‘Use Urban Heritage for Recovery/Build Back Better’ (Step-by-Step Guide):

- First: Understand the HBUR process with all its components (entity groups and domains) and phases.
- Second: Set up a team to design the HBD process (a small group of people with different views and backgrounds is ideal).
- Third: Transfer the abstract representations in the metamodel (entity groups and domains) to specific entities that are relevant in the specific case. For example, fill in the abstract term ‘decision makers’ with specific decision-making bodies and persons in the case at hand.
- Fourth: Define key points and general objectives.
- Fifth: Select stakeholders.
- Sixth: Identify decision makers and governance structures.
- Seventh: Define a road map for the HBUR process that includes milestones, timing, and decisions.
- Eight: Implement the HBUR process and monitor it constantly.

The listed steps were developed by Ripp (2021) and have been adapted for this specific case.

### Pitfalls and potential problems

The greatest danger when using the metamodel for HBUR processes is not understanding the phases and progressing through them too quickly, for example, moving to the development phase without establishing a common understanding of the situation at hand. Neglecting the thorough preparation of the process (scoping phase) is another risk, which is particularly pertinent in postrecovery settings with urgent issues to be addressed. The weak scoping of development processes, which are fundamental to urban heritage, can also pose a significant threat to their overall success (Ripp 2021), and the same is true for HBUR processes. Therefore, investing the time and effort for a thorough preparation will ultimately pay off.

## Conclusions

The metamodel for HBD can potentially be used in postcrisis settings for HBUR in the way that has just been described. The principal elements of HBUR processes are similar to those of HBD processes. HBUD and HBUR take place within the same system of urban heritage and therefore share the same elements (see Fig. 4), which are represented in the metamodel by entity groups and domains. While the individual objectives in a setting are always unique to the specific situation and context, the five phases of the metamodel remain valid. Moreover, the emphasis on the preparation of HBUR processes is similar to the scoping of HBD processes. The next step would be to test the metamodel in a postcrisis setting.

This paper discussed the potential transfer of a metamodel for HBUD to a postcrisis urban recovery scenario. We demonstrated how the metamodel for HBUD can also potentially be used for HBUR in two phases (SHELTER project phases of response and recovery/build back better). It is beyond the scope of this paper to go into more detail on the systemic nature of cultural heritage and the complexities of its role in these processes, which changes throughout different phases of disasters and crises. Regardless, cultural heritage is much more than a collection of objects that need to be protected. A focus on processes and the entire system of urban heritage can change perceptions of it from something that needs to be protected to something that can also contribute to recovery.

To encourage this perspective, heritage must be understood in a holistic, systemic way together with its values for local communities and other users. These values, which go far beyond classical preservation values, hold the key to unlocking the role of cultural heritage in recovery. The metamodel for HBUD is a methodology or toolbox by which this can be done, and its use can ensure that the elements of successful processes are present and that relevant phases are followed. The metamodel can also express the complexity of the heritage and heritage system at hand and help to integrate all the relevant elements (subjects, objects, processes, and values) in the recovery process. It can be used to scope HBUR processes based on experiences from successful HBUD projects. The five phases of the metamodel can assist in designing and structuring HBUR projects. For this purpose, the metamodel needs to be applied and filled in with specific entities of the specific case and environment. The application of the HBUR scenario in a real-world situation with the metamodel should be the subject of further research.

A systemic understanding of cultural heritage took a prominent place on the international stage with the Recommendation on the Historic Urban Landscape (HUL) in 2011 (UNESCO 2011). However, the HUL document is still closely connected to the safeguarding and the protection of cultural heritage in an urban setting, using the new HUL approach. Defined specific steps for the implementation of the HUL were developed with the recommendation but were ultimately excluded from the formal recommendation and used only as an introduction to the recommendation. Several years after the recommendation, these steps have been elaborated in the HUL Guidebook, which mainly aimed just to disseminate the HUL approach. This model has undoubtedly promoted the role of urban heritage for sustainable urban development. Diverse cultural contexts have been considered in its development through a number of expert meetings in a global context, and a number of cases where it has been implemented have been published (Bruin Veldpaus, and Roders 2013; Veldpaus and Pereira Roders 2013; Pereira Roders and Bandarin 2019). The HUL model can be used to describe HBUD (Ripp 2021), but from a practical perspective, it does not provide enough detail to be applied in real-world settings and has not been developed with enough scientific rigour to develop the full potential of a metamodel. However, the development of a metamodel was not the objective of the HUL Guidebook. The number of published cases in which the HUL model has been applied indicates that there are challenges in its application, which is normal for any model. However, despite its limited application, the HUL approach shares the underlying systemic worldview of metamodelling, as the HUL recommendation was also developed using the theory of urban morphology, which focuses on connections and processes in the development of urban form (Conzen 2004).

It is important to note that the HUL recommendation and the described metamodel are not truly comparable. The Recommendation on the Historic Urban Landscape needs to be viewed and contextualised in the moment and time when it was developed, resulting from a long process of discussions, meetings, and case studies involving several experts. At that time, it was a major innovation, mainly because it helped to shift the view of urban heritage from a collection of buildings, built heritage, or ensemble to the more holistic concept of the HUL that included functions, use, and other layers (Ripp 2021, 130f). The major change initiated by the HUL recommendation was its emphasis on how different parts or entities and functions are *connected* and have contributed through a complex interplay (Ripp 2021, 25f) to the historic urban landscape that we can experience, use, develop, and destroy today. It was not meant to be a model that is to be practically used. Conversely, the metamodel discussed in this paper was developed with a specific use case as a starting point, and it takes into account all relevant elements of successful processes rather than only proposing different steps.

Because of the metalevel on which the metamodel is viewed, there are many other theories and concepts the relevance of which can be discussed in the future. One such example is the concept of authenticity, which could be related to the metamodel entity group ‘values”. Discussion of these concepts is not within the scope of this paper but would be a fruitful objective for further research.

The metamodel for HBUD is also an example of the Conservation 3.0 perspective, as it starts with the adaptive reuse of historic buildings in a way that can work as a vehicle for the recovery of society in postcrisis scenarios and can contribute to sustainable development in the long run. In Conservation 3.0, conservation is considered not a cost but rather an investment that is expected to lead to future returns in the form of social, environmental, and economic synergy effects for society as a whole. This means that conservation can be understood as a contributor to sustainable development and to recovery in postcrisis periods. The current global health crisis can be viewed as a test of our conceptions and understanding of resilience. We will probably be better prepared for a similar crisis in the future, but the next crisis that we face will undoubtedly be different.

## Data Availability

This paper is a based on desk and literature research. The data used comes mainly from Matthias Ripp’s and Christer Gustafson’s PhD theses, as well as from the reports of COMUS, HERO, SHELTER and Halland Model projects. The datasets used and/or analysed during the current study are available from the corresponding author on reasonable request. All data generated or analysed during this study are included in this published article and its supplementary information files.

## Abbreviations

COE: Council of Europe
COMUS: Community-Led Urban Strategies in Historic Towns (COE/OWHC) project
DRM: Design research methodology
EC: European Commission
GT: Grounded theory
HBUD: Heritage-based urban development
HerO: Heritage as Opportunity (URBACT II Project)
HUL: Historic Urban Landscape
MM: Metamodel
PTF: Preliminary technical file
OWHC: Organisation of World Heritage Cities
THRIVE: Metamodel for Heritage-Based Urban Development (based on meTamodel HeRItage deVElopment)
UNESCO: United Nations Educational, Scientific and Cultural Organisation

## Footnotes

1 https://culture.ec.europa.eu/resources/coronavirus-response

2 https://pro.europeana.eu/event/towards-recovery-digital-capacity-building-in-the-cultural-heritage-sector

3 https://www.heritagefund.org.uk/funding/closed-programmes/culture-recovery-fund-for-heritage-0

4 See: Council of Europe (2016), City of Regensburg, M. Ripp, and N. Scheffler (2011) and Gustafsson (2009)

5 https://www.google.com/search?q=urban+resilience+definition&rlz=1C5GCEM_enSE976SE976&oq=Urban+resilience&aqs=chrome.1.69i57j0i512l4j0i22i30l5.3057j0j7&sourceid=chrome&ie=UTF-8

6 https://openknowledge.worldbank.org/handle/10986/27396

7 Fusco Girard et al. 2020.

8 Considering the analogies of heritage-based urban development and heritage-based urban recovery as elaborated on p. XXX, these points are also valid for HBUR.
